# Study of Degradation Profile and Development of Stability Indicating Methods for Cefixime Trihydrate

**DOI:** 10.4103/0250-474X.57295

**Published:** 2009

**Authors:** S. P. Gandhi, S. J. Rajput

**Affiliations:** Pharmaceutical Quality Assurance Laboratory, Pharmacy Department, Faculty of Technology & Engineering, M. S. University of Baroda, Vadodara-390 001, India

**Keywords:** Stress degradation, cefixime trihydrate, spectrophotometry

## Abstract

The degradation behavior of cefixime trihydrate was investigated under different stress conditions of acidic hydrolysis, alkaline hydrolysis and oxidation using spectrophotometry. Stability indicating spectrophotometric methods were developed that could separate the drug from its degradation products formed under these stress conditions. The UV spectral characteristics of the drug and degraded products were quite different and zero and first order derivative ultraviolet spectrophotometric methods were used to study the extent of degradation. Cefixime trihydrate was found to degrade extensively under experimental conditions. The methods were validated by establishing the linearity, inter and intraday precision, accuracy, selectivity and specificity.

The need to develop a stability indicating method using stress degradation has been recommended by International Conference of Harmonization[1]. The stress conditions should include the effect of temperature, humidity, light, oxidizing agents and susceptibility across a wide range of pH values. The aim of the current study was to study the degradation behavior of cefixime trihydrate (CEF) under a few ICH prescribed conditions and to develop validated stability indicating spectrophotometric assay methods.

CEF is an orally active third generation semi synthetic cephalosporin. Chemically, CEF is (6R,7R)-[[(Z)-2-(2-aminothiazol-4-yl)-2-[(carboxymethoxy)imino]acetyl]amino]-3-ethenyl-8-oxo-5-thia-1-azabicyclo [4.2.0]pet-2-ene-2-carboxylic acid trihydrate[2]. CEF is official in USP[2], BP[3] and EP[4]. The three pharmacopoeia describe ion pair HPLC method for estimation of cefixime from bulk drug and formulation. Only a slight change in the pH of mobile phase was perceived among the three techniques. Literature survey revealed that RP-HPLC method for determination of cefixime in biological fluids[5] and HPTLC method for determination of cefixime in presence of ceftriaxone and cefotaxime[6] are reported but these methods could not be used as stability indicating methods.

A Shimadzu 1601 UV/Vis spectrophotometer with matched 1 cm quartz cuvettes was used for the spectral measurements. The UVPC software was used for all the calculations. Silica gel 60F 254 precoated plates from Merck Ltd. were used for TLC studies. Standard bulk drug sample of cefixime trihydrate was provided as gift sample by Dalas Biotech Ltd., Alwar, Delhi. All other reagents used were of AR grade.

Stock solution of CEF (1000 μg/ml) was prepared in methanol. Suitable aliquots ranging from 0.01 to 0.25 ml were taken and diluted to 10 ml with water to give concentration in the range of 1 to 25 μg/ml. For all degradation studies, CEF at a concentration of 1 mg/ml was used. The degradation was done under strong, moderate as well as mild conditions. The samples were withdrawn initially and then after at regular time intervals. The degradation was checked using TLC. The aliquots withdrawn were suitably diluted with water to get the working solutions for spectrophotometric study.

For acid decomposition, drug solution was exposed to 0.01 M and 0.1 M HCl at 25°, 50° and 80° for up to 12 h. For alkaline decomposition, drug solution was exposed to 0.01 M and 0.1 M NaOH at 25°, 50° and 80° for up to 12 h. For oxidative decomposition, initial studies were performed in 1% H_2_O_2_ at 25, 50 and 80° for 8 h. Subsequently the drug was exposed to 3% H_2_O_2_ at these temperatures for 2 h.

For analysis of degraded samples, 1 ml of the degraded solution was withdrawn and diluted to 10 ml with water. The initial absorbance of the drug, at zero time was considered as 100% concentration and degradation was correlated with this concentration. To check decomposition, TLC was performed on Silica gel 60F 254 precoated plates using benzene: methanol: glacial acetic acid (1:1:1) as the mobile phase and spots were detected in an iodine chamber.

The zero order UV spectrum of CEF showed maximum absorbance at 287 nm. The solutions which were totally degraded by NaOH and H_2_O_2_ did not show any absorbance at this wavelength so decrease in absorbance was a direct measure of extent of degradation in partially degraded solutions (fig. 1). Though the spectral characteristics of acid degraded solution were quite different from the standard drug, the solution showed substantial absorption at 287 nm. The overlapped first order derivative curve (fig. 2) for both CEF and HCl degraded solution were obtained and zero crossing points for CEF and acid degraded solution were determined. CEF showed zero absorbance at 225 nm so this wavelength was used to estimate acid degraded solution and CEF was estimated at 242 nm at which the absorbance was zero for acid treated solution.

**Fig. 1:**
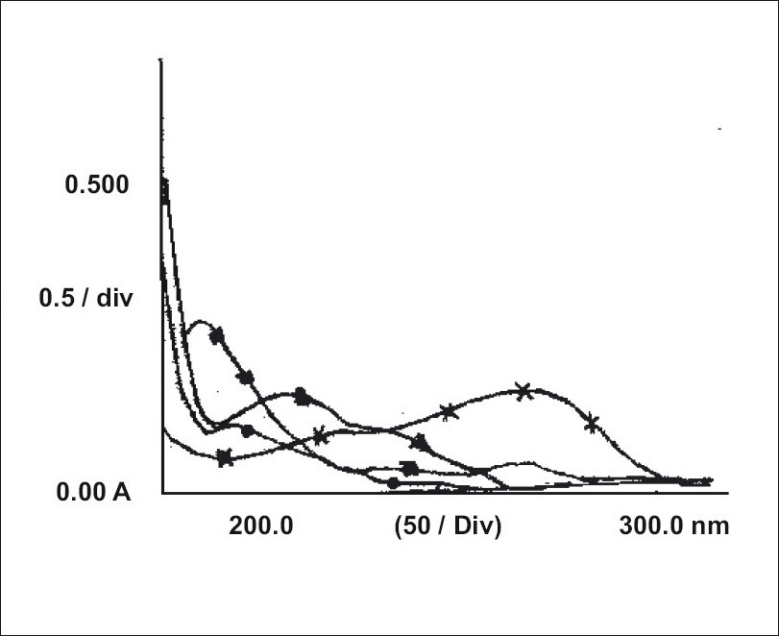
Overlapped zero order spectra of CEF and its degraded solutions. (×–×–×) shows the UV spectrum of untreated drug and (▲–▲–▲), (•–•–•) and (■–■–■) show the spectra of acid, alkali and peroxidetreated drug solutions, respectively. The spectra show that alkali and peroxide degraded solutions have no significant absorbance at the analytical wavelength of cefixime. Acid degraded solution interferes with the analysis.

**Fig. 2:**
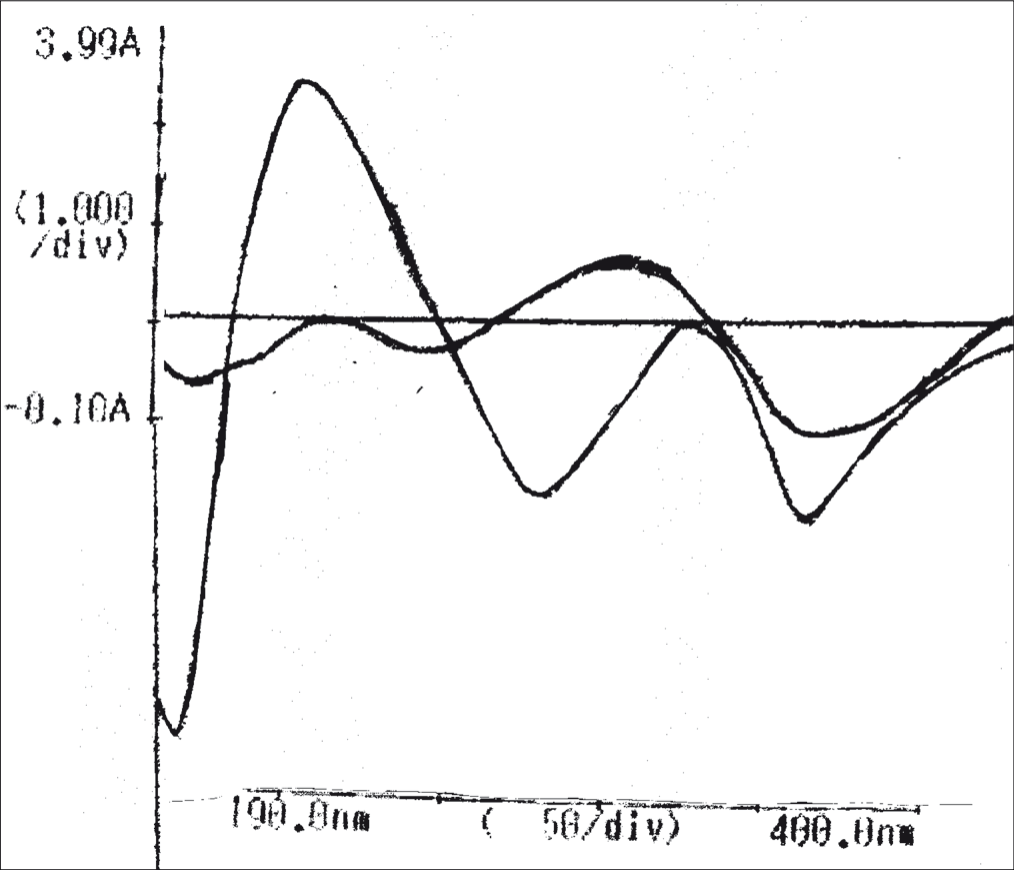
Overlapped first derivative spectra of cefixime and its acid degraded solution Cefixime was estimated at 242 nm, the zero crossing point for acid degraded solution.

Validation of the methods was done by studying various parameters. Linearity was studied by analyzing ten concentrations of the drug prepared in the methanol in the range of 4-24 μg/ml in triplicate and fitting the data into best fitted curve. Precision was verified by repeatability and intermediate precision studies. Repeatability was established by analyzing three different concentrations in triplicate on the same day whereas intermediate precision was checked by repeating the studies on different days. Accuracy of the method was tested by adding three concentrations of standard drug solution sequentially to a mixture of degraded solution and determining the recovery of the added drug. Specificity of the methods towards the drug was studied by analyzing a mixture containing standard drug and the stressed samples.

CEF has a lactam and amide linkage in its molecular structure making it amenable to acidic and basic hydrolysis. The lactam ring in cephalosporins is often opened during hydrolysis and the product formed may undergo side reactions like condensation[7] or dimerization[8] giving compounds totally different in their chemical properties. The TLC studies showed that Rf value for the standard drug was 0.48. The HCl and H_2_O_2_ treated solutions appeared at 0.68 and 0.70 respectively whereas the NaOH treated solution could not be detected. The spectroscopic studies of the stressed samples of CEF suggested following behavior of the drug under stress conditions.

It was observed that around 25% of CEF was degraded on heating at 80° for 1 h in 0.01 M NaOH. The drug was totally degraded if heated at 80° for 4 h in 0.1 M NaOH (fig. 3). The degradation was somewhat slower in acidic conditions as 25% drug was degraded if heated with 0.01 M HCl at 80° for 2.5 h. The 100% degradation in 0.1 M HCl was observed at 80° after 7 h (fig. 4). The degradation was very rapid under oxidative conditions, as 25% drug was degraded if left at 25° with 1% H_2_O_2_ in 3.5 h (fig. 5). Slight heating expedites the degradation procedure and the sample withdrawn just after 10 min of heating at 80° showed total degradation. Total degradation under all these conditions was confirmed by TLC. The analysis of CEF and its degradation products formed under stress conditions was possible using spectrophotometric method.

**Fig. 3:**
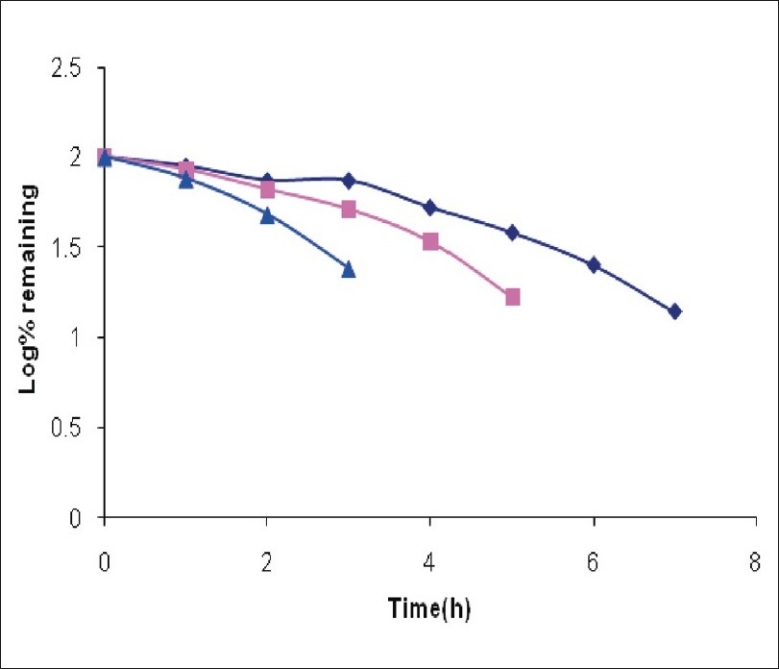
Degradation of cefixime under alkaline conditions (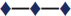) Represents degradation profile of cefixime in 0.1 N NaOH at 25°, (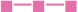 degradation profile of cefixime in 0.1N NaOH at 50° and (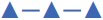 Degradation profile of cefixime in 0.1 N NaOH by reflux at 80°

**Fig. 4:**
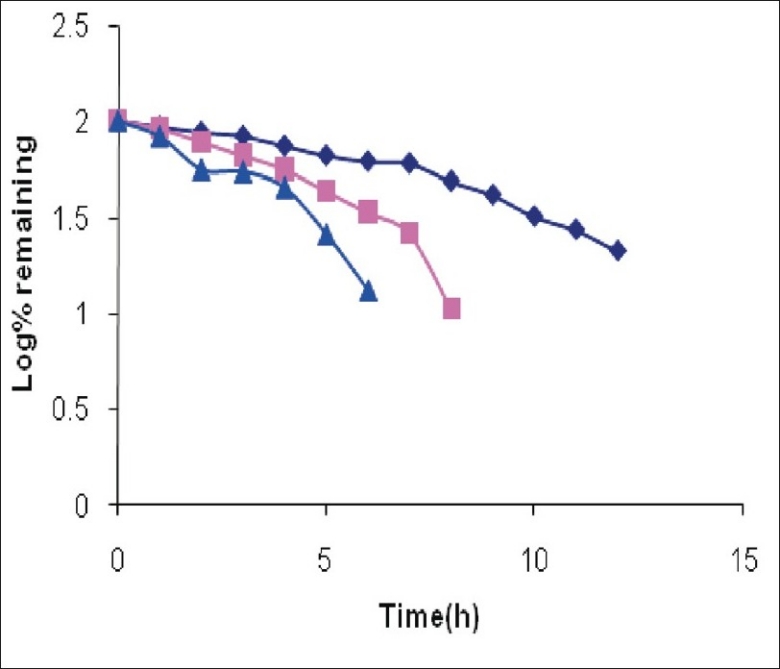
Degradation of cefixime under acidic conditions (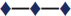 Represents degradation profile of cefixime in 0.1 N HCl at 25°, (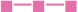 degradation profile of cefixime in 0.1 N HCl at 50° and (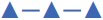 degradation profile of cefixime in 0.1 N HCl by reflux 80°

**Fig. 5:**
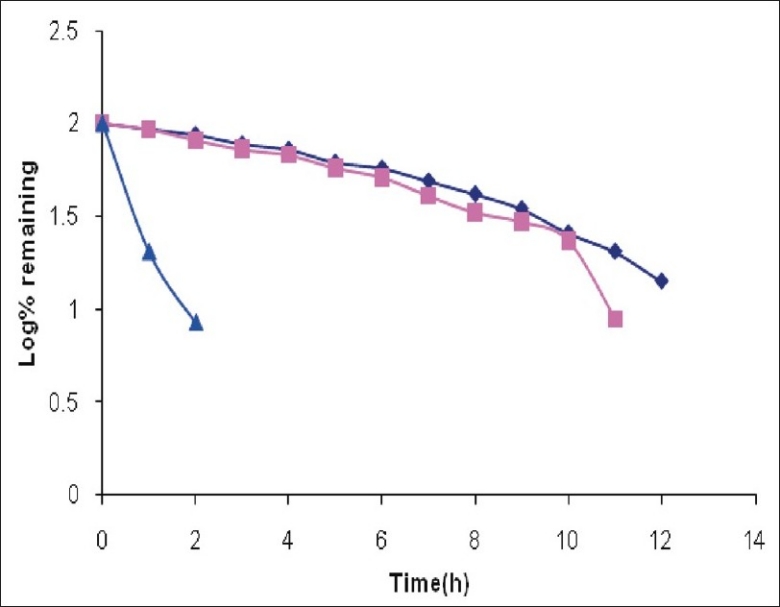
Degradation of cefixime under oxidative conditions (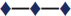) Represents degradation profile of cefixime in 1% H_2_O_2_ 25°, (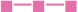 degradation profile of cefixime in 1% H_2_O_2_ at 50° and (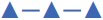 degradation profile of cefixime in 1% H_2_O_2_ by reflux at 80°

The spectral characteristics of the drug were totally different from its degradation products so zero order and first derivative UV method could be used to estimate CEF in presence of its degradation products. The result of validation studies showed that calibration curve was linear in the range of 4-24 μg/ml. The mean values for slope, intercept and correlation coefficient are reported in Table 1.

**TABLE 1:** OPTICAL PARAMETERS FOR SPECTROPHOTOMETRIC METHOD

Parameters	UV spectroscopy	First derivative spectroscopy
λ_max_	287 nm	242 nm
Beer's Range (μg/ml)	4 to 24	4 to 24
Regression Equation	Y=0.0408x−0.003	Y=0.0104x+0.0169
LOD (μg/ml)	0.0368	0.227
LOQ (μg/ml)	0.122	0.758
Correlation Coefficient (r^2^)	0.9999	0.9987
%RSD	0.299	0.597
Accuracy	98-100.4	98-102
Recovery	99.75-100.36	100.5-100.8

The values indicated here are the mean of three values.

The developed methods were found to be precise as indicated by low values (0.44% and 0.83%) for inter and intraday precision respectively. The accuracy was determined by recovery studies of spiked samples. The good recoveries (100±0.45) suggest method to be accurate. The study showed that CEF is susceptible to hydrolytic and oxidative degradations but the developed spectrophotometric method can be used as a stability indicating method to differentiate the drug from its degraded products. Though no attempts were made to identify and quantify the degraded products, the methods can be successfully used to determine the degradation of drug during storage.
